# AS-OCT–Based Biomarkers of Early Postoperative Bleb Morphology May Predict Surgical Success After PRESERFLO MicroShunt Implantation

**DOI:** 10.1167/tvst.15.10.47

**Published:** 2026-10-28

**Authors:** Tony Ishaq, Theresa Ishaq, Luise Zintl, Thomas Lehmann, Menelaos Papadimitriou, Daniel Meller, Somar Hasan

**Affiliations:** 1 Department of Ophthalmology, University Hospital Jena, Jena, Germany; 2 Institute of Medical Statistics, Computer and Data Sciences, University Hospital Jena, Jena, Germany; 3 Faculty of Medicine, Friedrich-Schiller University Jena, Jena, Germany; 4 Department of Ophthalmology, University Hospital Mannheim, Mannheim, Germany; 5 Mannheim Faculty of Medicine, University of Heidelberg, Mannheim, Germany

**Keywords:** glaucoma, Preserflo Microshunt (PMS), anterior segment optical coherence tomography (AS-OCT), biomarkers, surgical success

## Abstract

**Purpose:**

The purpose of this study was to identify early postoperative anterior segment optical coherence tomography (AS-OCT) biomarkers of bleb morphology that may predict surgical success following PRESERFLO MicroShunt (PMS) implantation in patients with glaucoma.

**Methods:**

Retrospectively, patients who underwent PMS implantation were included. AS-OCT imaging was performed 4 weeks after surgery. Qualitative (presence of conjunctival cysts and spaces, reflectivity of Tenon's capsule, and presence of an episcleral lake) and quantitative (maximal bleb length, height and width along with maximal episcleral lake length, height, and width) features were evaluated and surgical success 6 months after surgery was evaluated. Associations between these features and surgical success were analyzed.

**Results:**

Fifty-one eyes of 46 patients were included, 26 eyes (51%) met the success criteria for complete success (CS). Baseline characteristics were comparable between success and failure groups. The presence of an episcleral lake was significantly associated with later surgical success (odds ratio [OR] = 9.76, 95% confidence interval [CI] = 2.23–42.72, *P* < 0.01) and the maximum episcleral lake length (MLL), width (MLW), and height (MLH) were significantly higher in the success group compared to the failure group (3.18 ± 1.59 vs. 1.09 ± 1.63 mm, 3.04 ± 1.87 vs. 0.98 ± 1.4, 0.33 ± 0.2 vs. 0.15 ± 0.22, respectively, *P* < 0.05 for all). Lasso regression identified MLL and MLW as key predictors of success. A multivariable model incorporating these parameters achieved 79.1% accuracy (sensitivity 80.8% and specificity 77.3%) in predicting CS 6 months after the surgery.

**Conclusions:**

Early detection of an episcleral lake and its dimensions via AS-OCT may serve as a valuable predictor of surgical success following PMS. In contrast, conventional bleb parameters such as height, width, or wall reflectivity were not associated with outcomes.

**Translational Relevance:**

AS-OCT biomarkers could help predict surgical success following PMS.

## Introduction

Glaucoma is the second main cause of blindness after cataract and the first cause of irreversible blindness worldwide.^1^ Successful management of glaucoma would lower the risk of functional impairment and blindness.

Reduction of intraocular pressure (IOP) was proven to be the main treatment option that slows disease progression. Many surgical approaches have shown to effectively reduce IOP. Filtering surgery like trabeculectomy offers a significant and long-lasting reduction of IOP^2^^,^^3^ and is, after many decades of its first description by Cairns et al., still considered to be the gold standard of glaucoma surgery.^4^^,^^5^ Despite its effectiveness, trabeculectomy carries the risk of significant complications.^6^^,^^7^

The PRESERFLO MicroShunt (PMS; Santen Pharmaceutical Co., Ltd., Osaka, Japan) is a bleb forming device made of SIBS (Poly(styrene-block-isobutylene-block-styrene)) which can be implanted using an ab-externo approach and delivers significant IOP reduction with a good safety profile.^8^^–^^10^

Bleb scarring remains the principal cause of failure following filtering glaucoma surgeries with reported rates ranging from 9% to 43% after trabeculectomy^3^^,^^8^ and 21% to 29% following PMS,^8^^,^^11^ representing a persistent clinical challenge. A better understanding and assessment of bleb morphology during the early postoperative phase may provide critical insight into the wound healing and bleb maturation process and enable the application of prophylactic strategies before irreversible scarring occurs.^12^^–^^14^

Anterior segment optical coherence tomography (AS-OCT) is a noninvasive imaging modality that is increasingly used in the evaluation of bleb morphology.^15^^–^^17^ It provides high-resolution cross-sectional images of the bleb, which cannot be adequately visualized with traditional methods such as biomicroscopy or ultrasound. AS-OCT has thus significantly enhanced our understanding of bleb architecture.^18^^–^^21^ Importantly, AS-OCT has enabled the identification of early morphological features that may predict the long-term success or failure of the surgery. To date, most of these studies have focused on trabeculectomy.^22^^,^^23^ However, bleb morphology following PMS differs significantly from that observed after trabeculectomy,^18^ suggesting that prognostic biomarkers identified for trabeculectomy may not be applicable to PMS. The aim of this study was to identify predictive biomarkers of early postoperative bleb morphology using AS-OCT images following PMS implantation.

## Materials and Methods

This is a retrospective comparative study. We included eyes of patients who underwent PMS implantation as a stand-alone procedure. Inclusion criteria were the diagnosis of primary open angle glaucoma (POAG) or pseudoexfoliation glaucoma (PXG), age of ≥40 years old, and a minimum follow-up of 6 months. Exclusion criteria were previous intraocular surgeries (except non-complicated cataract surgery with IOL implantation in the bag), previous glaucoma surgeries, and any kind of ocular surgery affecting the conjunctiva.

Collected preoperative data included age, sex, glaucoma type, localization, IOP at surgical decision, and the number of glaucoma medications (NoMs). Postoperative data were collected at 6 months postoperatively and included date of surgery, date of examination, IOP, NoMs, and any postoperative complications or secondary interventions. In addition, an AS-OCT image of the whole bleb area was performed 30 (± 20) days following surgery using a predefined protocol as described below.

Bleb morphology was evaluated qualitatively and quantitatively.

Included eyes were classified 6 months post-surgery as success or failure, with success being defined as an 18≥IOP≥6 millimeters of mercury (mm Hg) with at least 20% reduction from the baseline IOP without (complete success [CS]) or with glaucoma medication not exceeding the NoM at baseline (qualified success [QS]). Bleb needling or open conjunctival revision as well as any kind of glaucoma surgery or glaucoma laser interventions were considered as failure. Reduction of visual acuity to hand motion and less was also considered as failure.

To account for correlation of multiple measurements (both eyes of 5 patients are investigated), continuous baseline and postoperative measurements of the whole study population were compared by linear mixed regression models. Continuous variables were summarized by means and standard deviation, and absolute and relative frequencies are provided for categorical variables.

Influence of preoperative parameters (age, sex, baseline IOP, and NoM) along with different qualitative or quantitative features of the bleb morphology on the postoperative success were examined using the generalized linear mixed model (GLMM) with binary link function, and odds ratios (ORs) with 95% confidence intervals (CIs) as well as *P* values are provided for each parameter. Variable selection for the multiple regression model was performed using least absolute shrinkage and selection operator (LASSO) regression. Predictive accuracy, sensitivity, and specificity were determined following 10-fold internal cross-validation.

The statistical analysis was performed using IBM SPSS Statistics version 24 (IBM Corp., Armonk, NY, 2016) and R software version 4.5.2 (R Core Team, Vienna, Austria, 2026).

### Surgical Technique and Postoperative Management

We applied the same surgical technique and postoperative management as described elsewhere.^18^^–^^21^ In this study cohort, no antimetabolites were used during the postoperative period.

### Bleb Examination Using AS-OCT

All bleb examinations were performed with a high-resolution swept-source AS-OCT (ANTERION; Heidelberg Engineering GmbH, Heidelberg, Germany), a standardized protocol was always followed.

During the examination the eyelid was gently lifted with a cotton swab and the patient was asked to look in a direction allowing maximal exposition of the bleb (e.g., inferotemporally for superonasal blebs). The imaging module was used while active eye tracking was deactivated. The length of the measured area was always set at 7.5 mm, the width was individually adjusted to cover as much as possible of the bleb area, the chosen width was then documented.

Two sets of scans, each one consisting of 45 single scans, were performed, the first one being tangential (Fig. 1a) and the second one radial (Fig. 1b), this resulted in a total of 90 scans of each bleb. All scans were independently evaluated by two examiners (authors T.I. and Th.I.), who performed both qualitative and quantitative assessments. Each examiner conducted the assessments independently and was blinded to the other examiner's findings. To evaluate intraobserver reliability, the first examiner repeated all qualitative and quantitative assessments after a sufficiently long interval to minimize recall bias, whereas the initial assessments of both examiners were used to assess interobserver reliability.

**Figure 1.**
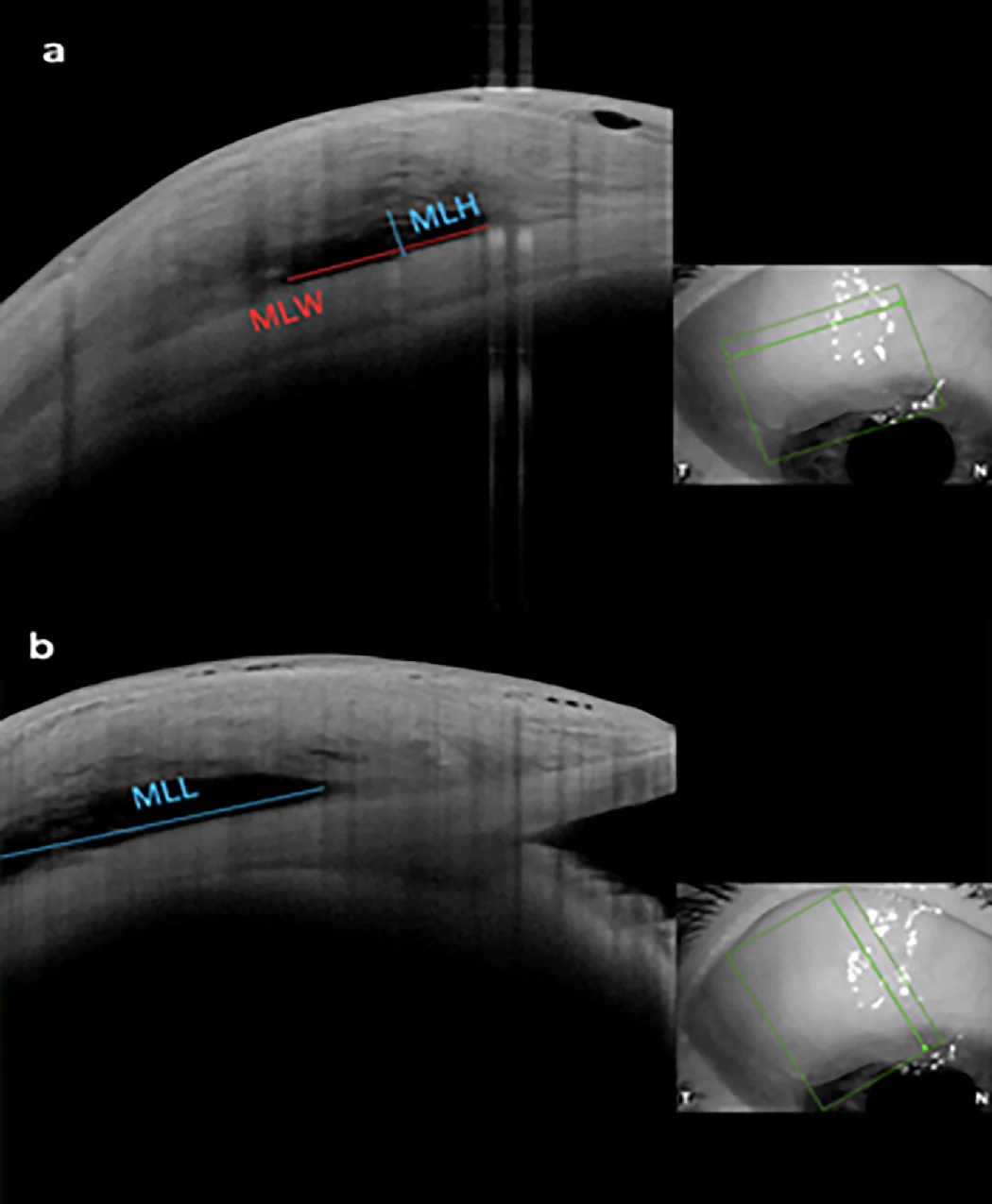
An AS-OCT scan of a filtering bleb after PMS implantation, the tangential (**a**) and radial (**b**) scan show the presence and dimensions of the episcleral lake. MLH, maximal lake height; MLL, maximal lake length; MLW, maximal lake width.

**Figure 2.**
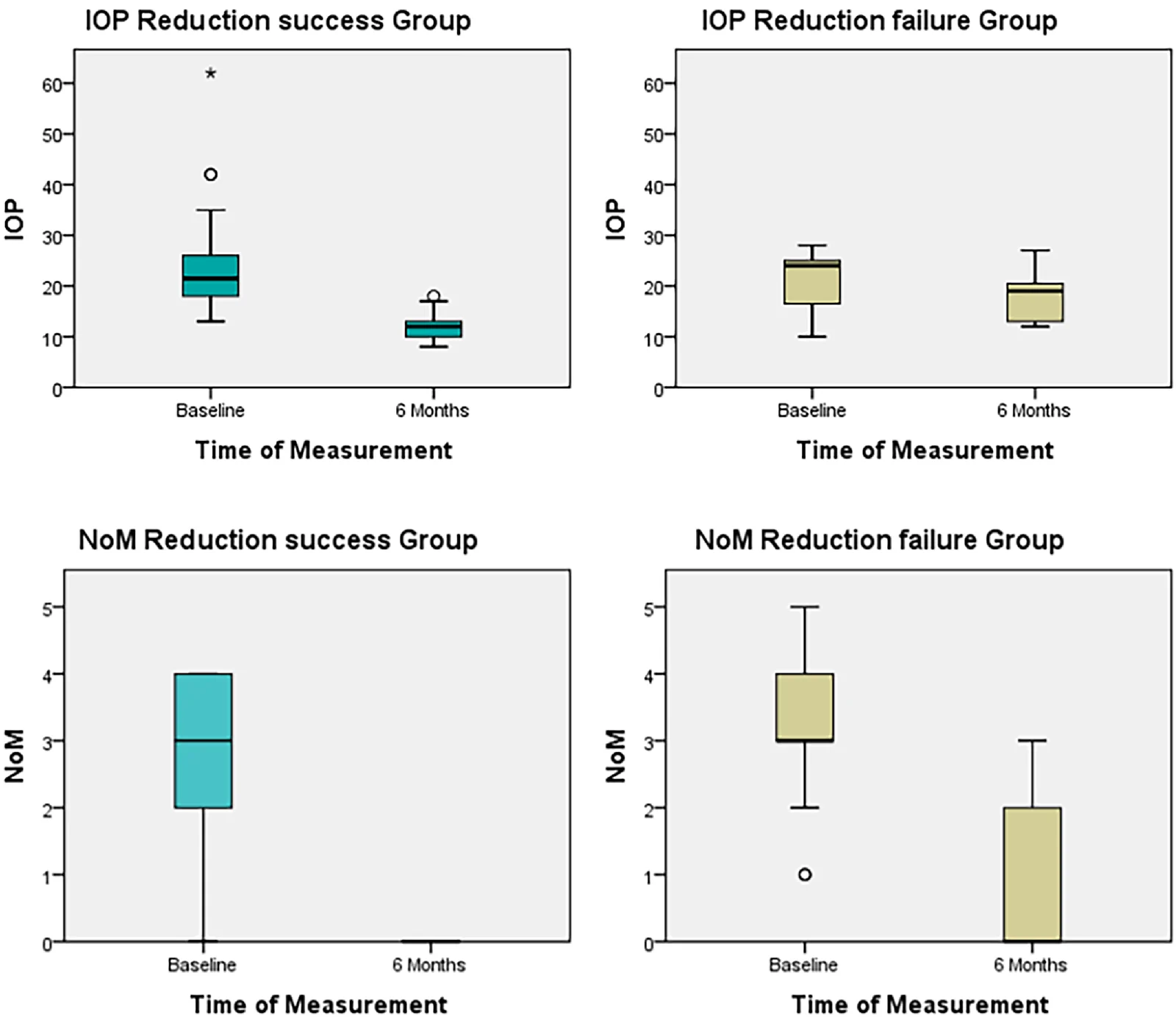
Changes in IOP and NoM for the CS group and failure group 6 months after surgery.

The qualitative assessment was performed based on the Jenaer Bleb Grading System including the determination of the presence/absence of conjunctival cysts, subconjunctival spaces, Tenon changes (hyporeflective, hyper-reflective, or cavernous changes) and/or episcleral lake. An episcleral lake was defined as the presence of an episcleral fluid filled space or cleft directly above the sclera and underneath tenon's capsule. These changes were compared to standard images which we published elsewhere.^24^

The following measurements were performed according to a previously defined protocol published elsewhere.^25^ These included maximal bleb height (MBH), maximal bleb length (MBL), and maximal bleb width (MBW),and, if existent, of the maximal lake height (MLH), maximal lake length (MLL), and maximal lake width (MLW) of the episcleral lake, along with the bleb wall thickness (BWT) and the distance to limbus (DtL). The 90 scans of each bleb were examined and the best fitting scan to each parameter was used for the measurements, commonly each of these parameters was measured using a different scan. Eyes that underwent needling or revision before receiving AS-OCT have been excluded from the study.

The study adhered to the Declaration of Helsinki guidelines. The study was approved by the Ethics Committee of Friedrich Schiller University Jena.

## Results

Fifty-one eyes of 46 patients (17 men and 34 women, mean age = 72.7 ± 7.5 years) were included. Preoperative data of CS/QS and failure groups are shown in Table 1. There were no significant differences between both groups regarding all preoperative parameters (*P* > 0.05 for all). CS and failure group showed a significant reduction of IOP (*P* < 0.03 for both groups) as well as of the NoM (*P* < 0.001 for both groups) at 6 months (Fig. 2). Patients classified as failure because of revision or second glaucoma surgery were censored from further analysis of postoperative IOP and NoM.

**Table 1.** Preoperative Data of the CS/QS and Failure Group

	All, *n* = 51	CS Group, *n* = 26	Failure Group, *n* = 25	*P* Value	QS Group, *n* = 29	Failure Group, *n* = 22	*P* Value
Age, mean ± SD	72.7 ± 7.5	74.42 ± 6.82	70.88 ± 7.83	0.06	74.03 ± 6.79	70.91 ± 8.11	0.14
Sex	Male *n* = 17 (33.3%)	Male *n* = 7 (26.9%)	Male *n* = 10 (40.0%)	0.23	Male *n* = 9 (31%)	Male *n* = 8 (36.3%)	0.76
Glaucoma diagnosis, POAG vs. PXG	POAG *n* = 39 (76.5%)	POAG *n* = 20 (76.9%)	POAG *n* = 19 (76%)	0.94	POAG *n* = 22 (75.8%)	POAG *n* = 17 (77.2%)	1.00
Lens status, phakic vs. pseudophakic	Phakic *n* = 29 (56.9%)	Phakic *n* = 12 (46.2%)	Phakic *n* = 17 (68%)	0.13	Phakic *n* = 13 (44.8%)	Phakic *n* = 16 (72.7%)	0.08
Laterality	Right eye *n* = 26 (51%)	Right eye *n* = 15 (57.7%)	Right eye *n* = 11 (44%)	0.34	Right eye *n* = 16 (55.1%)	Right eye *n* = 10 (45.4%)	0.57
Baseline IOP mm Hg, mean ± SD	23 ± 8.7	24.62 ± 10.97	21.32 ± 5.2	0.20	24.76 ± 10.39	20.68 ± 5.20	0.09
Baseline NoMs, mean ± SD	3.1 ± 1.2	2.77 ± 1.27	3.4 ± 1.04	0.08	2.86 ± 1.27	3.36 ± 1.05	0.14

IOP, intraocular pressure; NoMs, number of medications; POAG, primary open angle glaucoma; PXG, pseudoexfoliation glaucoma.

Preoperative data of the CS and failure group.

At 6 months, the most common cause for failure was an IOP-reduction less than 20% (*n* = 13, 52%) followed by an IOP >18 mm Hg (*n* = 11, 44%), the use of IOP lowering medication (*n* = 10, 40%). and by bleb revision (*n* = 10, 40%). Criteria for revision was an IOP exceeding 21 mm Hg or the target pressure as defined by a glaucoma surgeon under maximal tolerated medication.

Analysis of preoperative parameters and success rates at 6 months:

- No significant influence was found of the following parameters on CS or QS at 6 months: age, sex, preoperative IOP, NoMs, lens status (phakic versus pseudophakic), glaucoma diagnosis (POAG versus PXG), or eye laterality (right versus left, *P* > 0.05 for all).

Analysis of bleb morphology in early postoperative phase and success rates at 6 months:

- The bleb images were obtained, on average, 29.1 ± 11.12 days for the CS group and 30.48 ± 8.37 for the failure group after the surgery (*P* = 0.61), the majority of these images (*n* = 41, 80.4%) were obtained between 15 and 45 days after the surgery.

Results of qualitative and quantitative parameters of early bleb morphology are summarized in Table 2 and Table 3. Agreement between observers of our assessment method was already tested und reported in a previous publication.^24^

**Table 2.** Results of the Qualitative Measurements

	All, *n* = 51	CS Group, *n* = 26	Failure Group, *n* = 25	*P* Value	QS Group, *n* = 29	Failure Group, *n* = 22	*P* Value
Conjunctival cysts	29 (56.9%)	13 (50%)	16 (64%)	0.32	14 (48.3%)	15 (68.2%)	0.25
Subconjunctival spaces	46 (90.2%)	24 (92.3%)	22 (88%)	0.61	27 (93.1%)	19 (86.4%)	0.64
Hyporeflective tenon changes	21 (41.2%)	11 (42.3%)	10 (40%)	0.87	12 (41.4%)	9 (40.9%)	1.00
Hyper-reflective tenon changes	3 (5.9%)	1 (3.8%)	2 (8%)	0.54	1 (3.4%)	2 (9.1%)	0.57
Cavernous tenon changes	6 (11.8%)	3 (11.5%)	3 (12%)	0.96	3 (10.3%)	3 (13.6%)	1.00
Present episcleral lake	34 (66.7%)	23 (88.5%)	11 (44%)	<0.01	25 (86.2%)	9 (40.9%)	<0.01

Results of the qualitative measurements of the whole study population. *P* values are derived from generalized linear mixed models.

**Table 3.** Results of the Quantitative Measurements

	All, *n* = 51	CS Group, *n* = 26	Failure Group, *n* = 25	*P* Value	QS Group, *n* = 29	Failure Group, *n* = 22	*P* Value
MBH	1.65 ± 0.49	1.62 ± 0.49	1.68 ± 0.5	0.67	1.64 ± 0.51	1.66 ± 0.46	0.85
MBL	7.71 ± 1.8	7.76 ± 1.8	7.67 ± 1.8	0.85	7.67 ± 1.75	7.77 ± 1.87	0.85
MBW	9.35 ± 1.6	9.6 ± 1.56	9.1 ± 1.6	0.28	9.47 ± 1.77	9.20 ± 1.34	0.55
MLH	0.24 ± 0.23	0.33 ± 0.2	0.15 ± 0.22	0.01	0.35 ± 0.24	0.12 ± 0.15	<0.01
MLL	2.15 ± 1.91	3.18 ± 1.59	1.09 ± 1.63	<0.01	3.17 ± 1.70	0.82 ± 1.25	<0.01
MLW	2.03 ± 1.94	3.04 ± 1.87	0.98 ± 1.4	<0.01	3.02 ± 1.89	0.73 ± 1.06	<0.01
BWT	1.1 ± 0.4	1.07 ± 0.43	1.2 ± 0.28	0.42	1.11 ± 0.43	1.13 ± 0.25	0.82
DtL	5.72 ± 1.5	5.67 ± 1.64	5.76 ± 1.3	0.84	5.66 ± 1.61	5.07 ± 1.30	0.75

MBH, maximal bleb height; MBL, maximal bleb length; MBW, maximal bleb width; MLH, maximal lake height; MLL, maximal lake length; MLW, maximal lake width; BWT, bleb wall thickness; DtL, distance to limbus.

Results of the quantitative measurements presented as mean ± SD in mm of the whole study population. *P* values are derived from generalized linear mixed models.

Intraobserver and interobserver reliability were assessed for the qualitative and quantitative measurements. Results are summarized in Table 4 and Table 5. Repeated evaluations by the same examiner demonstrated substantial to excellent agreement for the categorical variables, with Cohen's κ values ranging from 0.724 to 0.960. For continuous measurements, intraobserver reliability was good to excellent for most parameters, with intraclass correlation coefficients (ICCs) ranging from 0.788 to 0.942, except for MBW, which showed moderate reliability (ICC = 0.599).

**Table 4.** Results of the Intraobserver Reliability Tests for the Qualitative and Quantitative Measurements

	Number of Measurements	Cohen's κ/ICC	95% Confidence Interval	*P* Value
Conjunctival cysts	51	0.960	0.870–1.00	1.0^a^
Subconjunctival spaces	51	0.878	0.485–1.00	1.0^a^
Hyporeflective tenon changes	51	0.876	0.720–1.00	0.25^a^
Hyper-reflective tenon changes	51	0.847	0.00–1.00	1.0^a^
Cavernous tenon changes	51	0.898	0.606–1.00	1.0^a^
Present episcleral lake	51	0.909	0.762–1.00	0.5^a^
MBH	51	0.810	0.670–0.914	0.834^b^
MBL	51	0.823	0.665–0.919	0.761^b^
MBW	51	0.599	0.418–0.746	<0.01^b^
MLH	51	0.897	0.766–0.976	0.572^b^
MLL	51	0.931	0.856–0.974	0.126^b^
MLW	51	0.942	0.897–0.973	0.276^b^
BWT	34	0.922	0.795–0.983	0.191^b^
DtL	51	0.788	0.596–0.900	0.104^b^

Results of the intraobserver reliability tests for the qualitative and quantitative measurements. Test for systematic difference ^a^ exact Mcnemar test, ^b^ Wilcoxon signed-rank test.

**Table 5.** Results of the Interobserver Reliability Tests for the Qualitative and Quantitative Measurements

	Number of Measurements	Cohen's κ/ICC	95% Confidence Interval	*P* Value
Conjunctival cysts	51	0.152	0.00–0.332	<0.01^a^
Subconjunctival spaces	51	0.391	−0.072–0.779	1.0^a^
Hyporeflective tenon changes	51	0.876	0.724–1.00	0.25^a^
Hyper-reflective tenon changes	51	0.105	0.00–0.244	<0.01^a^
Cavernous tenon changes	51	0.622	0.156–0.912	1.0^a^
Present episcleral lake	51	0.909	0.762–1.00	0.5^a^
MBH	51	0.809	0.672–0.910	0.004^b^
MBL	51	0.722	0.505–0.861	0.113^b^
MBW	51	0.645	0.449–0.787	<0.01^b^
MLH	51	0.864	0.703–0.970	0.712^b^
MLL	51	0.895	0.808–0.955	0.894^b^
MLW	51	0.951	0.904–0.978	0.777^b^
BWT	34	0.929	0.812–0.978	0.789^b^
DtL	51	0.689	0.512–0.809	0.062^b^

Results of the interobserver reliability tests for the qualitative and quantitative measurements. Test for systematic difference ^a^ exact Mcnemar test, ^b^ Wilcoxon signed-rank test.

Interobserver reliability of the quantitative measurements was moderate to excellent, with the highest agreement observed for MLW (ICC = 0.951), BWT (ICC = 0.929), and MLL (ICC = 0.895). Interobserver agreement for qualitative measurements was more variable. The presence of an episcleral lake and hyporeflective Tenon changes demonstrated very good agreement, whereas conjunctival cysts and hyper-reflective Tenon changes showed only slight agreement.

GLMMs were fitted associating each qualitative and quantitative parameter and surgical success or failure. The results are presented in Table 6^7^,^8^.

**Table 6.** Results of the Generalized Linear Mixed Model of the Qualitative and Quantitative Measurements

	Odds Ratio for CS	95% Confidence Interval CS	Odds Ratio for QS	95% Confidence Interval QS
Conjunctival cysts	0.56	0.17–1.78	0.43	0.13–1.42
Subconjunctival spaces	1.65	0.23–11.43	2.15	0.3–15.09
Hyporeflective tenon changes	1.10	0.35–3.47	1.01	0.32–3.24
Hyper-reflective tenon changes	0.46	0.03–5.77	0.357	0.02–4.48
Cavernous tenon changes	0.96	0.16–5.50	0.72	0.12–4.22
Present episcleral lake	9.76	2.23–42.72	9.03	2.24–36.3
MBH	0.78	0.24–2.53	0.90	0.27–2.94
MBL	1.03	0.74–1.42	0.97	0.70–1.34
MBW	1.22	0.84–1.78	1.11	0.77–1.60
MLH	65.06	2.466–1716.25	757.57	12.1–47426.79
MLL	2.1	1.37–3.22	2.52	1.54–4.12
MLW	2.1	1.34–3.29	2.54	1.49–4.34
BWT	0.45	0.06–3.28	0.83	0.10–6.71
DtL	0.96	0.65–1.42	0.94	0.63–1.39

Results of the generalized linear mixed model of the qualitative and quantitative measurements.

**Table 7.** Results of the Multiple Generalized Linear Mixed Model With MLL and MLW

	Odds Ratio for CS	95% Confidence Interval	*P* Value
MLL	1.58	0.89–2.80	0.111
MLW	1.46	0.83–2.56	0.179

CS, results of the multiple generalized linear mixed model with maximal lake length (MLL) and maximal lake width (MLW) as independent variables.

**Table 8.** Results of the Multiple Generalized Linear Mixed Model With MLL, MLW, and MLH

	Odds Ratio for Success QS	95% Confidence Interval	*P* Value
MLL	1.78	0.87–3.62	0.111
MLW	1.54	0.68–3.48	0.291
MLH	1.10	0.01–518.0	0.975

QS, results of the multiple generalized linear mixed model with maximal lake length (MLL), maximal lake width (MLW), and maximal lake height (MLH) as independent variables.

The presence of an episcleral lake, its maximum length, maximum width, and maximum height showed an increased probability of surgical success at 6 months. None of the other morphological parameters demonstrated a statistically significant association with surgical success (*P* > 0.05 for all).

Variable selection among all candidate parameters was performed using LASSO regression to identify predictors of surgical success at 6 months. MLL and MLW of the visible episcleral lake were retained as significant predictors. ORs with 95% CIs from the final multivariable GLMM are shown in Table 7.

Internal cross-validation demonstrated that a multivariable GLMM using these 2 predictors achieved 79.1% accuracy in forecasting surgical success (CS) at 6 months postoperatively. The model's sensitivity was 80.8% and specificity 77.3%.

Maximum length, maximum width, and maximum height were identified as predictors of qualified success at 6 months using LASSO regression. ORs with 95% CIs for these predictors are presented in Table 8. Using these 3 parameters, the model achieved an accuracy of 81.0% for predicting QS, with a sensitivity of 86.2% and a specificity of 75.8%.

## Discussion

Scarring is the primary cause of bleb failure in filtering surgeries.^14^ Identifying prognostic factors may help recognize eyes at higher risk and enable prophylactic interventions to improve success. Evaluating bleb morphology in the early postoperative period could provide valuable insights into the ongoing scarring process, potentially serving as an indicator of mid- and long-term outcomes.

Several morphological parameters have already been identified as prognostic indicators following trabeculectomy. For instance, Khamar et al. found that bleb walls with multiform reflectivity, multiple internal layers, and microcysts were associated with an increased likelihood of success at 6 months.^22^ Similarly, Kokubun et al. identified early bleb wall reflectivity as a predictor of bleb outcome.^23^ Nakano et al. also found that blebs with uniformly reflective wall and few hyporeflective areas 2 weeks post-surgery were associated with bleb failure at 6 months.^26^ Early bleb wall reflectivity after trabeculectomy was also found to have a predictive value in patients with neovascular glaucoma.^27^ The width of filtration opening measured 2 weeks following trabeculectomy was identified as a prognostic factor for surgical success 12 months after surgery.^28^ Several other studies showed an association between early morphological features such as the reflectivity of the bleb wall after trabeculectomy and subsequent IOP control.^29^^,^^30^

Emerging surgical techniques in filtering surgery using stent-based approaches have led to less invasive procedures and an improved safety profile. Compared to traditional trabeculectomy, stent-based filtering surgery results in a significantly different bleb morphology.^18^^–^^20^ For instance, resulting blebs following PMS tend to exhibit fewer conjunctival microcysts, thicker bleb walls, and a shallower but more posteriorly extending episcleral lake.^18^ These morphological differences suggest that prognostic parameters derived from trabeculectomy may not directly apply to PMS.

In our study, the implantation of PMS achieved a significant IOP reduction 6 months after surgery along with reducing the NoM, findings that are similar to those reported in other studies.^8^^,^^9^ Fifty-one percent of the patients met our CS criteria, which is consistent with the outcomes reported by Baker et al. in their randomized controlled trial.^8^

When evaluating qualitative parameters of bleb morphology using AS-OCT, the presence of an episcleral lake emerged as a highly significant predictor of CS and QS, whereas other parameters did not show prognostic value. Examining the quantitative parameters, the maximum length and width of this lake showed a significant association with CS and QS, while its maximum height showed a significant correlation only with QS. Other morphological parameters, such as MBH, MBL, or MBW, distance to limbus or bleb wall thickness did not correlate with the surgical outcome at 6 months.

Barberá et al.^31^ reported similar findings in their study, noting that the early presence of a fluid cavity and multilayered Tenon-conjunctiva was predictive of bleb success 1 year after surgery. They also observed that blebs with little or no fluid around the tube were associated with higher IOPs at 1 year, this also comes in accordance with our results regarding higher success rates by increasing length and width of the episcleral lake.

The importance of a visible episcleral lake after PMS might be explained by the surgical technique as the localization of the stent is assured to be in the episcleral space. The two-step closure of the tenon and conjunctiva ensures an episcleral drainage after PMS compared with a mixed episcleral / intratenon drainage following trabeculectomy. This might have made the intratenon and intraconjunctival changes less relevant following PMS in the early postoperative period.

Other morphological features—including overall bleb dimensions, BWT, distance to the limbus, and conjunctival cysts or spaces— did not demonstrate significant correlations with surgical success following PMS whereas some of these have shown prognostic value following trabeculectomy. We also did not find a significant association between the reflectivity of the tenon or the bleb wall and surgical success, these served as well as biomarkers for surgical success after trabeculectomy. It is possible, however, that these features might be indicators of success at later stages, as bleb morphology can change during maturation process.^31^^,^^32^ In fact, Barberá et al. observed that successful blebs at 1 year either had a moderate filtering surface with a thick, low-reflective wall or a large filtering surface with a thinner, more reflective wall.^31^ They also found that taller blebs and larger episcleral fluid cavities were associated with higher long-term success.

In this study, we did not assess the vascularization of the bleb which also has been shown to have a predictive value. Schneider et al. assessed bleb vessel density using AS-OCT angiography and found that a higher density was significantly associated with future bleb revisions.^33^

This study has several limitations: the retrospective design and the small cohort size represent significant limitations to the validity of the results. Additionally, all eyes included in this study had no prior intraocular or conjunctival surgeries—except for uncomplicated cataract procedures—and all were treated intraoperatively with Mitomycin C 0.2 mg/mL. Therefore, it remains uncertain whether these findings apply to eyes with previous surgeries or those treated with different Mitomycin C concentrations.

The assessment of the AS-OCT images was conducted by the same observer which might have resulted in observer bias. However, the collection and assessment of the images were performed prior to the collection of the clinical data, thereby ensuring that the observer was blinded to the clinical outcomes at the time of assessment. Additionally, the measurements were guided by reference images from previously published studies.^21^^,^^24^ Because this study relied on manually performed measurements, we conducted intraobserver and interobserver reliability analyses to provide a more transparent assessment of measurement reproducibility, despite the use of clearly defined parameters and a standardized imaging protocol. Overall, agreement was good to excellent for most parameters. However, greater variability was observed between different observers than between repeated assessments by the same observer, particularly for quantitative measurements. Automated image analysis may therefore represent an important methodological advancement for future studies by reducing observer-dependent variability and improving reproducibility.

It is important to note that, in some cases, the AS-OCT images did not capture the full extent of the bleb or the episcleral lake. Only the visible portions were measured; thus, posterior regions or areas obscured by the eyelid could not be assessed. This limitation may affect the validity and generalizability of the findings. It is also important to note that surgical success in this study was defined strictly as IOP between 6 and 18 mm Hg without (CS) or with glaucoma medication not exceeding the NoMs at baseline (QS). Therefore, the prognostic value of the identified morphological features may not be directly applicable to cases using different success criteria. On the other hand, the use of standardized surgical technique and a uniform imaging protocol along with the acquisition of multiple AS-OCT scans over the entire bleb area enhances the validity and reliability of our findings. Unlike most previous studies, which were typically limited to two to three cross-sectional scans, our comprehensive scanning approach minimizes the risk of overlooking relevant morphological details.

Given the exploratory nature of the analyses and the limited sample size, no formal adjustment for multiple comparisons was performed. Therefore, *P* values should be interpreted cautiously.

## Conclusions

This study underscores the utility of AS-OCT in evaluating bleb morphology after PMS during the early postoperative phase. It also demonstrates that the early presence of an episcleral lake along with higher length and width might serve as positive predictive factors for surgical success.
